# Cathepsin B Gene Disruption Induced *Leishmania donovani* Proteome Remodeling Implies Cathepsin B Role in Secretome Regulation

**DOI:** 10.1371/journal.pone.0079951

**Published:** 2013-11-14

**Authors:** Teklu Kuru Gerbaba, Lashitew Gedamu

**Affiliations:** Department of Biological Sciences, University of Calgary, Calgary, Alberta, Canada; Royal Tropical Institute, Netherlands

## Abstract

*Leishmania* cysteine proteases are potential vaccine candidates and drug targets. To study the role of cathepsin B cysteine protease, we have generated and characterized cathepsin B null mutant *L. donovani* parasites. *L. donovani* cathepsin B null mutants grow normally in culture, but they show significantly attenuated virulence inside macrophages. Quantitative proteome profiling of wild type and null mutant parasites indicates cathepsin B disruption induced remodeling of *L. donovani* proteome. We identified 83 modulated proteins, of which 65 are decreased and 18 are increased in the null mutant parasites, and 66% (55/83) of the modulated proteins are *L. donovani* secreted proteins. Proteins involved in oxidation-reduction (trypanothione reductase, peroxidoxins, tryparedoxin, cytochromes) and translation (ribosomal proteins) are among those decreased in the null mutant parasites, and most of these proteins belong to the same complex network of proteins. Our results imply virulence role of cathepsin B via regulation of *Leishmania* secreted proteins.

## Introduction

Leishmaniasis is a spectrum of diseases ranging in severity from spontaneously healing and non-healing cutaneous lesions (caused by several species; *L. tropica, L. major, L. aethiopica and L. mexicana*) to fatal visceral disease (caused by *L. donovani* complex species; *L. donovani*, *L. infantum*, *L. chagasi*). There are over 500,000 cases of visceral leishmaniasis with 50,000 deaths annually. There is no vaccine against any form of leishmaniasis, available drugs have serious side effects, and drug resistant cases are on the rise [1]–[5].

*Leishmania* papain family cysteine proteases, cathepsin B and cathepsin L, are potential drug targets and vaccine candidates. *Leishmania* genome consists of multi copy Cathepsin L and a single copy cathepsin B cysteine protease genes [6]–[8]. The importance of *L. mexicana* cathepsin L and B cysteine proteases has been studied using null mutant parasites in mice. *L. mexicana* cathepsin L null mutant parasites are unable to induce lesion development in mice and protect mice against challenge with wild type parasites [9]. Unlike *L. mexicana* cathepsin L, *L. mexicana* cathepsin B null mutants induce lesion development in mice although they show reduced survival inside macrophages *in vitro* [10]. *L. donovani* cathepsin B null mutant parasites have not been generated yet, and their pathogenesis *in vivo* and potential as an attenuated vaccine candidate remains to be determined. In a closely related protozoan, *Trypanosoma brucei,* RNAi targeting of cathepsin B leads to clearance of parasites from the bloodstream and prevents lethal infection in mice [11]. Enzymatic assays and sequence analysis suggest difference in the function of cathpesin B of different *Leishmania* species. *L. major* cathepsin B, unlike *L. mexicana* cathepsin B, has no activity against Z-Arg-Arg-AMC [12], which was attributed to difference in S_2_ sub-site amino acid [13], [14]. *L. major* cathepsin B has glycine at S_2_ sub-site, whereas *L. mexicana* cathepsin B has a serine. *L. donovani* cathepsin B, which has a glycine residue at S_2_ sub-site and 90% identical with *L. major* cathepsin B, shares only 60% identity with *L. mexicana* cathepsin B [8].

By using antisense inhibition and specific protease inhibitors, studies from our lab and others have shown the importance of *L. donovani* cathepsin B for survival inside macrophages and activation of latent-TGF-β1 *in vitro*. *L. donovani* cathepsin B null mutant parasites have not been generated yet, hence the role of *L. donovani* cathepsin B and the mechanisms by which cathepsin B affect *Leishmania* intracellular survival *in vivo* remain unknown. Here, we report the generation of *L. donovani* cathepsin B null mutant parasites and cathepsin B gene disruption induced proteome remodeling. Proteome profiling of *L. donovani* cathepsin B wild type and null mutant parasites hints at virulence role of cathepsin B via regulation of *Leishmania* secreted proteins.

## Materials and Methods

### *Leishmania*, Cell lines, and Growth media

*Leishmania donovani* 1S2D was obtained from Dr. Greg Matlashewski, McGill University, Montreal, Quebec, Canada. *Leishmania* parasites were maintained in M199 medium. *Leishmania* selections were done on semi solid SDM-79 medium as described previously [15]. U937 cell lines were obtained from ATCC and maintained in a complete RPMI media.

### Enzymes, Peptides, Antibodies, Plasmids, and Kits

Restriction enzymes were purchased from GE Healthcare or Invitrogen. AMC (7-Amino-4-Methyl-Coumarin), Z-Arg-Arg-AMC, and Z-Phe-Arg-AMC were purchased from Peptides International. Anti *L. major* cathepsin B antibody was kindly provided by Dr. Judy Sakanari, Sonoma State University, California, USA. Peroxidoxin-4 antibody was raised in mice [16]. Anti α-tubulin antibody was purchased from SIGMA and secondary antibodies were purchased from R&D Biosystems. Homologous recombination (pX63Hyg and pX63Neo) and episomal expression vectors (pXGSAT, pXNeo) were kindly provided by Dr. Stephen Beverley, University of Washington, St. Louis, Missouri, USA. Geneticin and hygromycin B were purchased from Invitrogen and nourseothricin was purchased from Sigma. Genomic and plasmid DNA preparation and gel purification kits were purchased from QIAGEN. DIG-High Prime DNA Labeling and Detection Starter Kit I was purchased from Roche Diagnostics. Western and southern blotting membranes and ECL western blot detection kits were purchased from GE Healthcare.

### DNA preparations

Mid log stage *Leishmania* parasites were washed three times with PBS and DNA was extracted using DNAeasy Blood and Tissue kit (QIAGEN). Small-scale plasmids were prepared by using Spin Mini Prep Kit (QIAGEN) and large-scale plasmids were prepared by using Maxi Prep Kit (QIAGEN).

### Generation of null mutant and complemented null mutant parasites

*L. donovani* cathepsin B null mutants were generated by deleting part of cathepsin B coding sequence (529 bp) containing active site cysteine and occluding loop (**Figure S1**). Knockout constructs were made by PCR amplifying and cloning 5' (819 bp) and 3' (765 bp) sequences flanking the 529 bp region. The 5′ flanking sequence was amplified with primers carrying *Hind*III and *Xho*I restriction sites (5′flankF, 5′-CTGCACAAGCTTTTGCAACfGTGCCCGCCGACGTG-3′; 5′flankR, 5′-CACCTCCTCGAGGCTCTTGCCGCTGACC-3′). The 3′ flanking sequence was amplified with primers carrying *Sma*I and BglII restriction sites (3′flankF, 5′-AGCGGTCCCGGGGATGGATCTGGTCAAGTACAAGG-3′; 3′flankR, 5′-AGCGGTAGATCTTTCTCAGCCTCCTTTCCCG-3′). The PCR amplified products were digested with restriction enzymes and gel purified. The purified PCR products were ligated with digested, dephosphorylated, and gel purified pX63-HYG and pX63-NEO plasmids. For complementation, cathepsin B coding sequence was amplified from pXNeo vector carrying a cDNA clone of *L. donovani* cathepsin B by using BamHI restriction site carrying primers (Forward, 5′-CTAGATGGATCCGATGGCTACTCCTG-3′; Reverse, 5′-GGCAGTGGATCCATGGCCCTCCGCGCCAAG-3′). The amplified product was gel purified and ligated into the *BamH*I site of digested, dephosphorylated, and gel purified pXGSAT vector. Ligation products were transformed into competent *E. coli* DH5α cells. *E. coli* DH5α competent cell preparations [17] and transformations [18] were done following standard procedures. Plasmids were isolated and insert sequences and orientations were confirmed by sequencing both strands of DNA at the University of Calgary UCDNA sequencing facility. The knockout constructs were linearized with *Hind*III and *Bgl*II restriction enzymes and the hygromycin construct was transfected into wild type parasites. The transfected parasites were plated on SDM-79 media containing 100 µg/ml hygromycin to select for heterozygous mutants. The single allele knockout parasites were transfected with the neomycin knockout construct and plated on SDM-79 media containing 100 µg/ml hygromycin and 50 µg/ml geneticin to select for homozygous mutants. Cathepsin B complemented null mutants were created by transfecting pXGSAT plasmids carrying cathepsin B coding sequence into the null mutant parasites. The transfected null mutant parasites were plated on SDM-79 media containing 100 µg/ml hygromycin, 50 µg/ml geneticin and 100 µg/ml nourseothricin. *Leishmania* log stage promastigotes (4×10^7^ parasites) were transfected with 5 µg linearized knockout construct DNA and 30 µg circular plasmid DNA as previously described [19]. Transfected parasites were re-suspended in drug-free media for 24 hours then drugs were added for selection. The generated double allele knockouts and complemented null mutant parasites were characterized by using PCR and southern and western blot analysis.

### Southern blotting

Probe labeling and detections were done using DIG-High Prime DNA Labeling and Detection Starter Kit I (Roche Applied Bioscience). For DIG labeling, the following target sequences were PCR amplified by using specific primers: cathepsin B 529 bp region (forward, 5′-CTCGAGGAGGTGCGCAAGCTGATGGG-3′; reverse, 5′-GTAGATGGTGGTGTTCGGGCAGGGCGGG-3′), LdCys2 (cathepsin L) probe (forward, 5′-ACCACTGGCAACATCGAAGGCCAG-3′; reverse, 5′-GCTGGGCTCCGGCTAGGCCGCTGTCG-3′), sat/nourseothricin marker (forward, 5′-CCCTCTCGAGATGAAGATTTCGGTGATCCC-3′; reverse, 5′-CTCGCCCCGGGTTAGGCGTCATCCTGTGCTCC-3′), hygromycin (forward, 5′-CCCTCTCGAGATGAAAAAGCCTGAACTCAC-3′; reverse, 5′-CTCGCCCCGGGCGGGCTATTCCTTTGCCCTCGGA-3′), and neomycin (forward, 5′-CCCTCTCGAGATGGGATCGGCCATTGAACA-3′; reverse, 

5′-CTCGCCCCGGGTCAGAAGAACTCGTCAAGA-3′).

The PCR products were gel purified and 1 ug of the purified products were labeled with DIG. *Leishmania* genomic DNA was digested overnight with *Pst*I enzyme. Ten micro gram of the digested DNA were run on 0.8% agarose gel and transferred onto positively charged nylon membranes. Blots were detected with anti-DIG antibody solution (75 mU/ml).

### Leishmania lysate preparations

*Leishmania* parasites were washed twice in ice-cold PBS and re-suspended in 5×10^8^ parasites per 500 µl lysis buffer. Two different lysis buffers were used to prepare lysates for protease assay (20 mM Tris pH 8.0, 10 mM EDTA, pH 8.0, 4 mM NaCl) and for western blot analysis (5 mM Tris pH 8.0, 2 mM EDTA pH 8.0, 0.5 mM PMSF). Lysates were kept on ice for 30 minutes, sonicated (3 cycles, 30 sec/cycle, 30 sec cooling in between; 45 mv), and centrifuged at 13400 xg for 20 minutes to remove cell debris. Protein concentrations were determined using BCA method and stored at -80^o^C until use.

### Western blotting

*Leishmania* protein lysates were run on SDS-Polyacrylamide gel and transferred on to Hybond P membranes (GE healthcare). ECL advanced western blot detection kit (GE healthcare) was used to detect proteins using primary antibodies and HRP conjugated secondary antibodies.

### Cysteine protease activity assay

*Leishmania* lysates were incubated with fluorogenic substrates (Z-Arg-Arg-AMC or Z-Phe-Arg-AMC) in a protease assay buffer (100 mM sodium acetate, pH 5.5, 10 mM dithiothreitol, 0.1% Triton X-100, 1 mM EDTA and 0.5% DMSO; pH 6.0) in 96 well plate at 37°C for 10 minutes. AMCs liberated from fluorogenic substrates by protease activity were quantified by ELISA reader at 460 nm emission and an excitation wavelength of 355 nm.

### Infection of U937 macrophages

Infections of U937 macrophages were done as previously described [20]. Cells were grown to confluency, washed, and cultured at a concentration of 2.5×10^6^ cells/cm^2^/well in a chamber slide in the presence of 150 nM PMA, and they were infected with stationary stage *Leishmania* parasites at a cell to parasite ratio of 1:20. Extracellular parasites were removed by washing in a complete RPMI media and infection was monitored by Diff-Quick (Baxter) staining.

### *Leishmania* proteome profiling

**Protein extraction.** *L. donovani* cathepsin B wild type and null mutant proteins were extracted from stationary stage parasites as described previously [21] and transported to UVic Genome BC Proteomics Centre (University of Victoria, Victoria, BC, Canada) for iTRAQ based LC-MS/MS analysis.

**Trypsin digestion and peptide labeling.** Trypsin digestions and iTRAQ labelings were done following standard procedures (Applied Biosystems, Foster City, CA, USA). Protein samples (100 µg each) extracted from cathepsin B wild type and null mutant parasites were precipitated overnight in acetone at 4^o^C followed by resolublization in 0.5 M triethylammonium bicarbonate (TEAB) and 0.2% SDS. The proteins were reduced with tris-(2-carboxyethyl) phosphine (TCEP), alkylated with methyl methanethiosulfonate (MMTS), and digested with trypsin (Promega). Peptdies of the wild type and the null mutant parasites were labeled with different iTRAQ reagents and combined for analysis with strong cation exchange (SCX) high performance liquid chromatography (HPLC) and liquid chromatography-tandem mass spectrometry (LC-MS/MS).

**SCX-HPLC and LC-MS/MS Analysis.** Peptides were fractionated by SCX-HPLC on a VISION Workstation (Applied Biosystems) equipped with Polysulfoethyl A SCX column, 100 mm (length)×4.6 mm (inner diameter), 5 um (particle size), and 300Å (pore size; Poly LC, Columbia, MD). Samples in buffer A (10 mM KPO_4_ (pH 2.7), 25% ACN) were injected onto a pre-equilibrated column under a flow rate of 0.5 ml minute^−1^. A gradient of 0-35% buffer B (10 mM KH_2_PO_4_, 25% ACN, 0.5 M KCl) in 30 minutes was applied. Peptide fractions were analyzed by reverse phase (RP) LC-MS/MS system. LC separations were done on a 75 µm×15 cm C18 PepMap Nano LC column (3 µm particle size, 100Å pore size, LC Packings, Amsterdam, The Netherlands) with A 300 µm×5 mm C18 PepMap guard column (5 µm particle size, 100Å pore size, LC Packings, Amsterdam, The Netherlands). Solvent A [water/acetonitrile (98:2, v/v), 0.05% formic acid] was used for sample injection and equilibration on the guard column at a flow rate of 100 µl minute^−1^. Solvent B [acetonitrile/water (98:2, v/v), 0.05% formic acid] was used for high-resolution chromatography and introduction into mass spectrometer at a flow rate of 200 nl minute^−1^.

Tandem mass spectrometry was performed on a hybrid quadrupole-time of flight (TOF) mass spectrometer (QStar Pulsar I) equipped with a nano-electrospray ionization source (Proxeon, Odense, Denmark). LC-MS/MS data acquisitions were done as described previously [21], [22]. Briefly, A 1 second TOFMS scan of mass range 400–1200 amu and two 2.5 second product ion scans of mass range 100–1500 amu were performed. The two most intense peaks over 20 counts with 2–5 charge state were selected for fragmentation. Fragmentation of the same isotopic cluster peaks was prevented by using a 6 amu (atomic mass unit) window. Ions selected for fragmentation were excluded from identification for 180 seconds. Protein identifications were done by searching the data against the *L. infantum* v.3.0 database (http://www.genedb.org/genedb/linfantum/ ) using ProteinPilot Version 2.0 [23] (Applied Biosystems). Proteins were considered to be confidently identified if they had an unused Prot Score > 1.3 (confidence ≥95%). ProteinPilot calculates relative abundance of a protein from average abundant values of all its peptides.

### Gene ontology (GO) enrichment and Biological network analysis

To determine over represented gene ontology terms, enrichment analysis was performed using a cytoscape plugin BiNGO Version 2.44 [24]. Modulated protein sequences were used to retrieve their respective yeast orthologs (Table S3) from *Saccharomyces cerevisiae* S288c (taxid:559292) refseq database by using BLASTP 2.2.25 [25]. BLASTP was run using default parameters and *Leishmania* sequence IDs that found hit with E ≤ 4.4E-02 and bit score of 32.7 were used for enrichment analysis in BinGO. Hypergeometric test (p < 0.05) with Benjamin-Hochberg FDR correction, whole annotation as a reference set, and *S. cerevisiae* as a test organism were used for the analysis. Interaction networks were generated by using Cytoscape MiMI plugin version 3.1[26] and well-connected interactions were identified by MCODE [27].

## Results

### Characterization of *L. donovani* cathepsin B null mutant parasites

**Figure 1:**
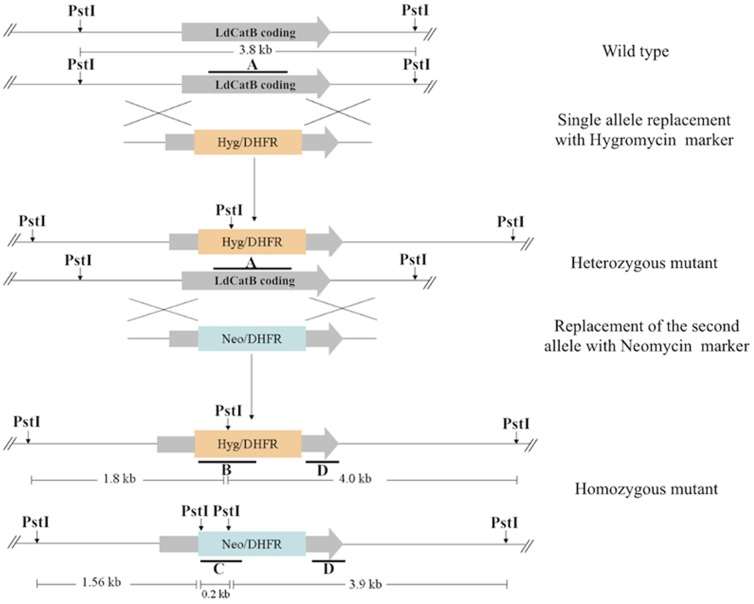
Homologous recombination based gene deletion strategy. Single allele cathepsin B mutant parasites were generated using hygromycin replacement construct and the remaining single allele was replaced with the neomycin construct. A, B, C, and D indicate probes used to screen the generated mutant parasites. ‘A’ binds to part of the mature region of *L. donovani* cathepsin B replaced by the hygromycin and neomycin markers. ‘B’ and ‘C’ are hygromycin and neomycin coding region probes respectively. ‘D’ hybridizes to the 3’ flanking sequences of the homologous recombination constructs.

**Figure 2:**
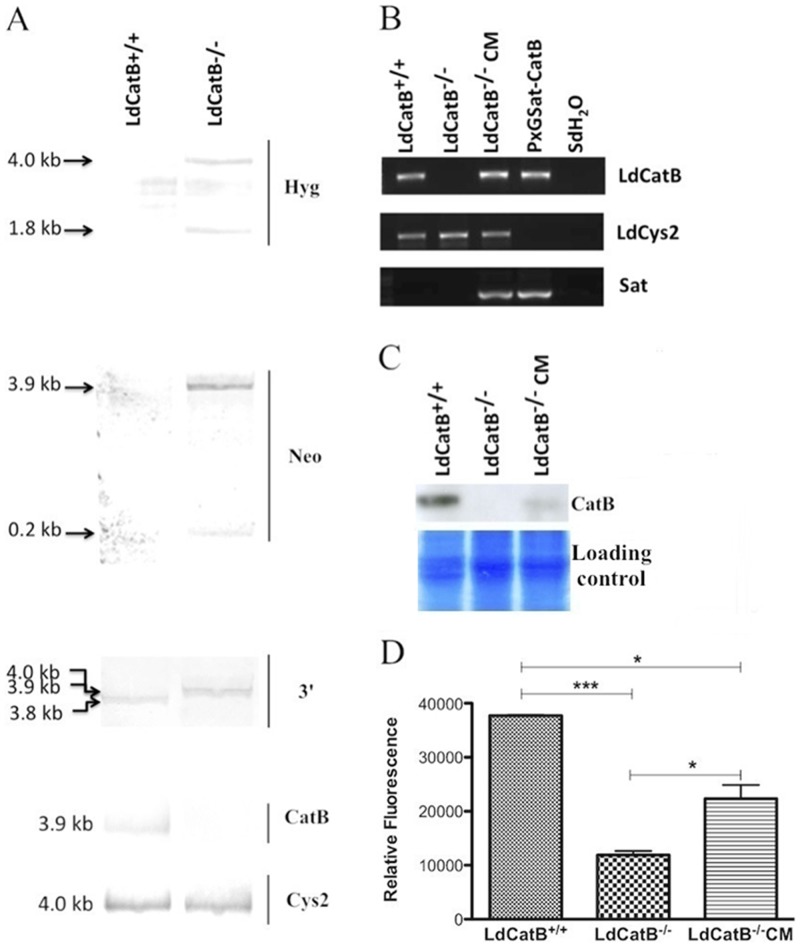
Characterization of *L. donovani* cathepsin B null mutant parasites. (**A**) Southern blot analysis of null mutant parasite (*L. donovani* cathepsin B^−/−^) using probes hybridizing to hygromycin (Hyg**),** neomycin (Neo), 3′ flanking sequence (3′), and deleted region of cathepsin B (Figure 1). (**B**) PCR characterization of null mutant and complemented null mutant parasites using specific primers of the deleted region of cathepsin B (LdCatB), cathepsin L sequence (LdCys2, loading control), and nourseothricin coding sequence (Sat). (**C**) Western blot detection of cathepsin B (CatB) protein. A 30-kDa band was detected in the cathepsin B^+/+^ and cathepsin B^−/−^CM parasites but not in the cathepsin B^−/−^ parasites by anti-*L. major* cathepsin B (1:5000 dilution) antibody. Commassie stained proteins were used as a loading control. (**D**) *L. donovani* cathepsin B cysteine protease readily cleaves Z-Phe-Arg-AMC. Parasite lysate (0.01 µg/µl) and Z-Phe-Arg-AMC (10 µM) were used for activity assay. Bars and error bars indicate the mean fluorescence and standard deviation of enzymatic activity of lysates prepared from three independent experiments. Statistical significance was determined by using paired t test. ***, P<0.0001; *, p<0.05. LdCatB^+/+^, *L. donovani* cathepsin B^+/+^; LdCatB^−/−^, *L. donovani* cathepsin B^−/−^; LdCatB^−/−^ CM, *L donovani* cathepsin B^−/−^ carrying episomal cathepsin B; pXGSat-CatB, vector carrying the open reading frame of cathepsin B.

**Figure 3:**
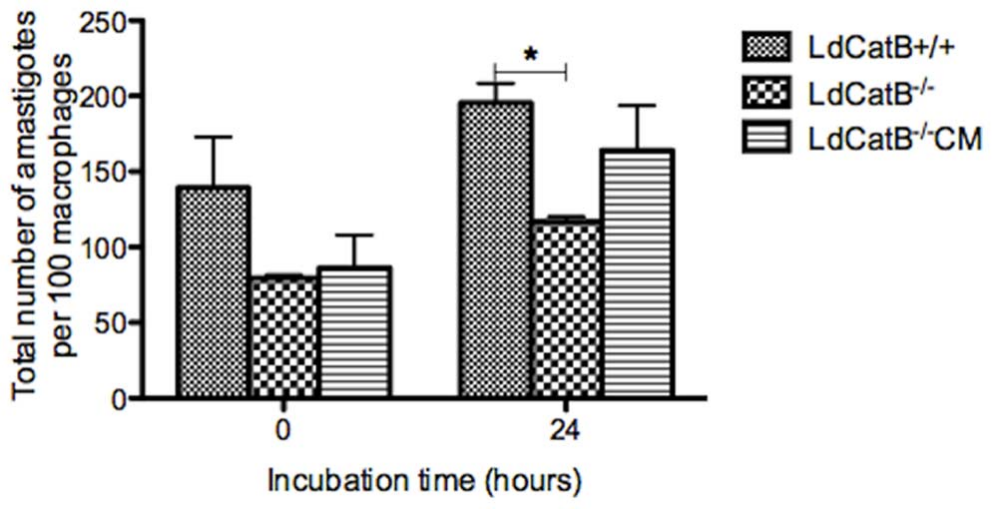
Intracellular survival role of *L. donovani* cathepsin B. *L. donovani* parasites were incubated with U937 macrophages for 6 hours, extracellular parasites were washed and incubated for 0 and 24 hours in fresh media. *L. donovani* cathepsin B^−/−^ parasites show a significantly reduced survival at 24 hours of incubation and complementation (cathepsin B^−/−^CM) increases the survival of the null mutant parasites. Infected macrophages were giemsa stained and intracellular amastigotes per 100 macrophages were counted. The error bars show the standard deviations of a triplicate experiment. *, p<0.05 determined using t-test.

We generated *L. donovani* cathepsin B null mutant parasites by deleting 529 bp region of the coding sequence containing the active site cysteine residue and the occluding loop **(** **Figure 1** **).** We confirmed proper integration of knockout constructs to the cathepsin B locus by southern blotting **(** **Figure 2** **)**. Hygromycin **(** **Figure 2A** **, Hyg)** and Neomycin **(** **Figure 2A** **, Neo)** probes hybridize only to the null mutant parasite DNA resulting in two expected size bands. The 3′ flanking region probe hybridizes to a 3.8 kb band in the wild type parasite and to 3.9 and 4.0 kb bands in the cathepsin B null mutant parasite (**Figure 2A** **,** **3**′). The 100 bp difference between the two bands is due to difference in a *Pst*I restriction site position on the neomycin and hygromycin markers. We confirmed cathepsin B gene disruption by a probe designed based on the 529-bp region of cathepsin B, which hybridizes only to the wild type parasite DNA (**Figure 2A**, **CatB**). Specific primers of the deleted region amplify only from the wild type and the complemented null mutant parasite DNA (**Figure 2B**, **LdCatB**). Specific primers of the nourseothricin marker confirm transfection of the episomal complementation plasmid **(** **Figure 2B** **, Sat)**. Cathepsin B 30-kDa mature protein was detected in the wild type and the complemented null mutant parasites, but not in the null mutant parasites (**Figure 2C**, **CatB**). We studied functional effect of cathepsin B disruption by testing protease activity and intracellular survival of the wild type and the null mutant parasites. *L. donovani* cathepsin B wild type lysates show significantly higher activity against Z-Phe-Arg-AMC, and complementation partially reverses protease activity of the null mutant parasites **(** **Figure 2D** **)**. We found no difference in the activity of *L. donovani* cathepsin B wild type and null mutant parasites against Z-Arg-Arg-AMC (data not shown). Cathepsin B null mutant parasites show significantly reduced number of amastigotes inside macrophages 24 hours after infection, and episomal complementation augments this effect **(** **Figure 3** **)**. The reduced number of amastigotes could be due to attenuated multiplication and/or survival of the parasites. There is no difference in the survival of the wild type and the null mutant parasites in culture (data not shown).

### Cathepsin B gene disruption modulates *L. donovani* proteome

**Figure 4:**
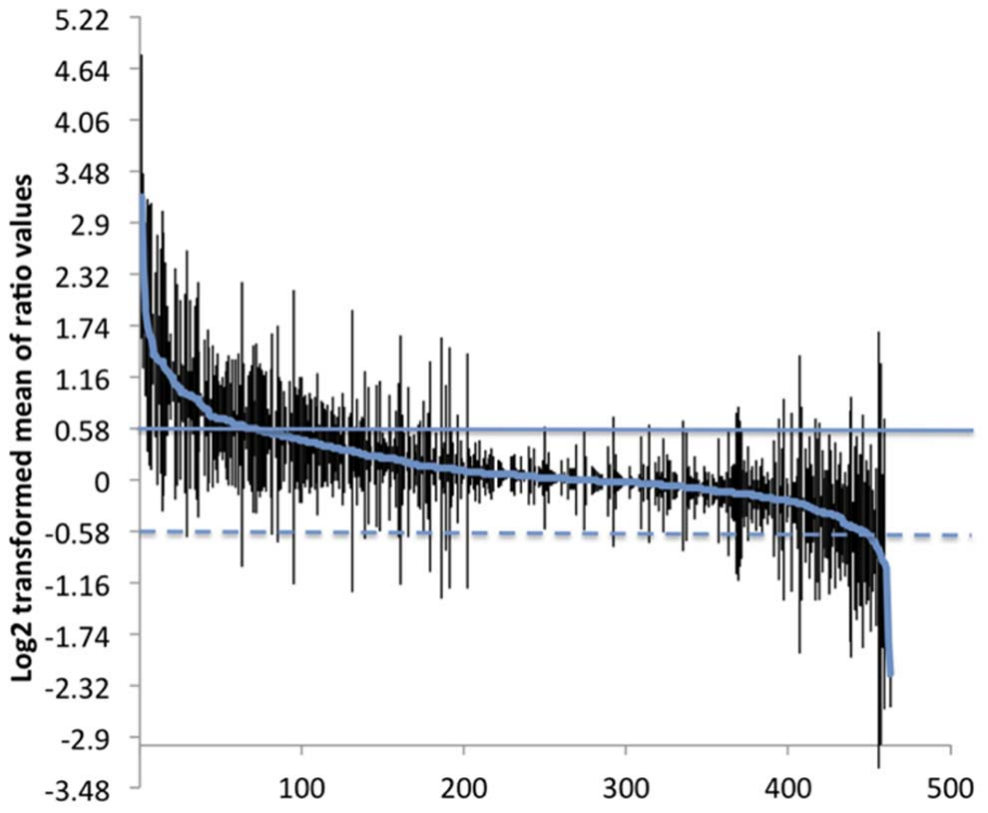
Cathepsin B gene disruption induced proteome remodelling. Log_2_ transformed mean of *L. donovani* cathepsin B wild type : *L. donovani* cathepsin B null mutant protein ratios and their standard deviations calculated from two independent experiments are shown. Mean of log_2_ transformed ratios above + 0.58 (increased by 1.5 fold change; solid horizontal line) are proteins considered to be increased in the wild type, whereas proteins below – 0.58 (decreased by 1.5 fold change; broken horizontal line) are proteins considered to be increased in the null mutant parasites. The proteins between horizontal lines were considered unchanged. The numbers on the X-axis are protein identities.

**Figure 5:**
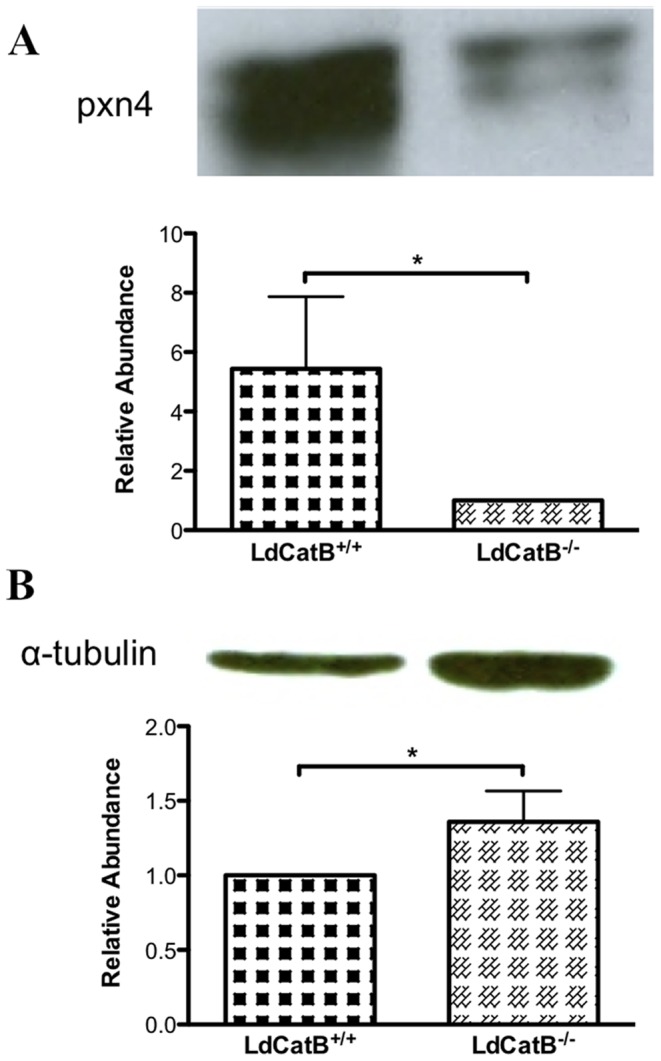
Validation of proteome data by western blotting. (A) Peroxidoxin-4 (pxn4) and (B) alpha tubulin (α-tubulin) western blot. Equal amounts (15 µg) of lysate proteins of 1×10^9^ *L. donovani* stationary stage parasites (*L. donovani* cathepsin B wild type, LdCatB^+/+^; *L. donovani* cathepsin B null mutant, LdCatB^−/−^) were used for detection by pxn4 (1∶200 dilution) and α-tubulin (1∶2500 dilution) antibodies. Histograms show average relative abundance of proteins determined by densitometry analysis with standard deviations from three independent experiments. Densitometry analysis of western blot bands was done using ImageJ software, and relative abundance was calculated by normalizing either to the wild type or null mutant parasite protein level. Statistical significance was determined by using Mann-Whitney test; *, *P* < 0.05.

*L. donovani* cathepsin B wild type and null mutant protein samples pooled from three independent preparations were analyzed using iTRAQ based quantitative proteomics and data generated from two experiments by iTRAQ swapping (iTRAQ 115 and iTRAQ 116) are presented here. Proteins detected in both experiments and represented by at least one unique peptide with ≥ 95% confidence were considered identified by LC-MS/MS. This resulted in the identification of a total of 463 proteins. The list of peptides used for identifications are given in **Table S1**. Each protein was identified on average by 8 peptides, and only four proteins were identified by a single unique peptide. Over 95% of the peptides had precursor delta masses less than 0.16 Da. The relative abundance of the 463 proteins was used to determine cathepsin B disruption induced modulation of *L. donovani* proteome (**Table S2**, **Figure 4**). Among the 463 proteins, 83 proteins were modulated at least by 1.5 fold change (**Table 1**). Sixty-five proteins were increased in the *L. donovani* cathepsin B wild type and 18 proteins were increased in *L. donovani* cathepsin B null mutant parasites. Cathepsin B is the most significantly modulated protein with 9.12 fold increase in the wild type parasites (**Table 1**). The level of cathepsin B detected in the null mutant parasites comes from peptides belonging to the non-deleted region of cathepsin B. Western blotting of peroxidoxin-4 (LinJ.23.0050), which was increased by 1.6 fold in the wild type parasites, and alpha tubulin (LinJ.13.1460), which was increased by 1.73 fold in the null mutant parasites, validate the proteome data (**Figure 5**). The fold change for peroxidoxin obtained by western blot is larger than that obtained by iTRAQ analysis. This is possibly due to underestimation of protein ratios by iTRAQ [28]–[31].

**Table 1:** Proteins modulated by *L. donovani* cathepsin B gene disruption.

ID	Description	Log_2_(WT/KO)^a^	Secretion^b^
LinJ.29.0860	Cathepsin B	3.19	
LinJ.21.0310	hexokinase	2.35	S
LinJ.05.0350	trypanothione reductase	1.98	AS
LinJ.29.1250	tryparedoxin	1.91	
LinJ.08.1220	hypothetical protein	1.74	S
LinJ.35.0990	aldose 1-epimerase	1.62	
LinJ.35.1670	60S ribosomal protein L26	1.61	
LinJ.24.2200	hypothetical protein	1.46	S
LinJ.05.0830	methylthioadenosine phosphorylase	1.41	S
LinJ.14.1240	enolase	1.40	AS
LinJ.32.0440	60S ribosomal protein L17	1.34	
LinJ.25.1160	aldehyde dehydrogenase	1.34	S
LinJ.29.2580	60S ribosomal protein L13	1.33	S
LinJ.14.0920	calpain-like cysteine peptidase	1.32	
LinJ.35.0410	40S ribosomal protein S3a	1.30	AS
LinJ.15.1070	glutamate dehydrogenase	1.24	S
LinJ.26.2290	nitrilase	1.22	AS
LinJ.26.1220	heat shock protein 70-related protein	1.19	AS
LinJ.26.0780	glutathione peroxidase-like protein	1.16	
LinJ.23.0860	3-ketoacyl-coa thiolase-like protein	1.14	S
LinJ.26.0610	10 kDa heat shock protein	1.10	S
LinJ.36.6110	hypothetical protein	1.07	
LinJ.35.0600	60S ribosomal protein L18a	1.06	S
LinJ.14.1450	myo-inositol-1-phosphate synthase	1.02	
LinJ.30.3000	glyceraldehyde 3-phosphate dehydrogenase	1.01	S
LinJ.13.1130	40S ribosomal protein S4	0.98	AS
LinJ.36.4100	S-adenosylhomocysteine hydrolase	0.97	S
LinJ.24.2170	40S ribosomal protein S8	0.97	S
LinJ.27.0510	calpain-like cysteine peptidase	0.96	S
LinJ.23.0220	endoribonuclease L-PSP (pb5)	0.96	
LinJ.28.2760	40S ribosomal protein S17	0.95	S
LinJ.26.1590	proline oxidase	0.94	
LinJ.02.0520	hypothetical protein	0.94	
LinJ.22.1410	40S ribosomal protein L14	0.92	S
LinJ.27.0190	proteasome alpha 7 subunit	0.91	
LinJ.30.3660	ATP synthase	0.90	S
LinJ.36.0800	hypothetical protein	0.87	AS
LinJ.15.1100	tryparedoxin peroxidase	0.84	
LinJ.25.2090	2,4-dihydroxyhept-2-ene-1,7-dioic acid aldolase	0.83	
LinJ.05.0960	dipeptidyl-peptidase III	0.80	AS
LinJ.26.1550	thimet oligopeptidase	0.79	AS
LinJ.35.3830	60S ribosomal protein L27A/L29	0.77	S
LinJ.21.2070	proteasome alpha 2 subunit	0.73	S
LinJ.36.4030	glycyl tRNA synthetase	0.71	AS
LinJ.36.1000	40S ribosomal protein S18	0.71	S
LinJ.31.0010	5-methyltetrahydropteroyltriglutamate-homocysteine S-methyltransferase	0.70	
LinJ.30.3390	60S ribosomal protein L9	0.69	S
LinJ.01.0440	ribosomal protein S7	0.69	S
LinJ.21.0980	hypoxanthine-guanine phosphoribosyltransferase (HGPRT)	0.69	S
LinJ.23.0050	peroxidoxin	0.68	S
LinJ.08.0330	mitochondrial associated ribonuclease	0.68	
LinJ.35.2270	kinetoplastid membrane protein-11 (KMP11-2)	0.68	S
LinJ.30.3790	60S acidic ribosomal protein P2	0.67	AS
LinJ.35.3840	60S ribosomal protein L23	0.66	S
LinJ.05.0510	ATPase alpha subunit	0.66	S
LinJ.27.2500	glycosomal phosphoenolpyruvate carboxykinase	0.66	S
LinJ.35.1900	60S ribosomal protein L36	0.63	S
LinJ.35.4790	glycine cleavage system H protein	0.63	
LinJ.14.0190	hypothetical protein	0.63	AS
LinJ.36.5870	isoleucyl-tRNA synthetase	0.63	AS
LinJ.32.0790	RNA binding protein	0.62	S
LinJ.16.1390	cytochrome c	0.61	S
LinJ.15.0320	ribonucleoprotein p18	0.61	S
LinJ.33.2520	heat shock protein	0.60	
LinJ.08.1010	hypothetical protein	0.60	S
LinJ.23.1190	hypothetical protein	-0.59	
LinJ.17.1380	hypothetical protein	-0.60	
LinJ.29.1190	hypothetical protein	-0.60	S
LinJ.35.1390	mitochondrial processing peptidase	-0.63	S
LinJ.28.1210	hypothetical protein	-0.64	
LinJ.06.0120	cyclophilin	-0.66	S
LinJ.34.0170	malate dehydrogenase	-0.69	
LinJ.24.1630	hypothetical protein	-0.70	
LinJ.10.1070	histone h3	-0.76	S
LinJ.07.0210	cytochrome c1	-0.80	
LinJ.13.1460	alpha tubulin	-0.80	S
LinJ.24.0870	triosephosphate isomerase	-0.85	S
LinJ.29.0810	U-box domain protein	-0.92	
LinJ.29.0890	high mobility group protein homolog tdp-1	-0.95	
LinJ.36.0210	elongation factor 2	-1.01	AS
LinJ.29.0950	ADP ribosylation factor 3	-1.08	
LinJ.29.0520	cofilin-like protein	-1.83	AS
LinJ.29.0790	lipophosphoglycan biosynthetic protein	-2.20	S

a Mean of log_2_ transformed wild type (WT) and knockout (KO) protein ratios from quantified in two independent experiments.

b Secreted (S) and actively secreted (AS) *L. donovani* proteins [32].

### Cathepsin B gene disruption modulates *Lesihmania* secreted proteins

*L. donovani* secreted proteins reported in [32] were used to examine the effect of cathepsin B disruption on *L. donovani* secretome. Sixty six percent (55/83) of the proteins modulated by *L. donovani* cathepsin B gene disruption were found to be *Leishmania* secretome proteins and 18% (15/83) of these were identified as actively secreted proteins [32].

### Gene ontology analysis of modulated proteins

**Figure 6:**
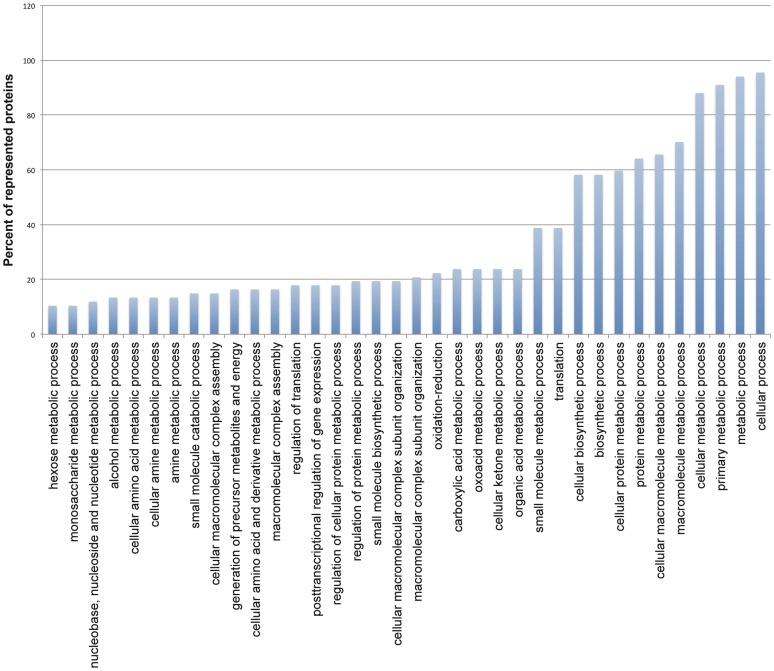
Biological process GO term enrichment analysis. Significantly (*p* value < 0.05) over represented biological processes are shown. P-values were determined by hypergeometric test after FDR correction. Percent of remodeled proteins represented in each cellular process are shown in bars. Non-redundant processes assigned to at least six (10%) modulated proteins are displayed.

In order to understand the functional category of the affected proteins, we analyzed functions enriched in the modulated proteins and found significantly enriched biological process GO-terms (**Figure 6** **).** The lists of the modulated proteins that represent the biological processes are given in **Table S4**. Translation (GO6412, 38.8%), oxidation-reduction (GO 55114, 22.3%), and macromolecular complex assembly (GO34622, 14.9%) are among the most highly enriched processes. Translation GO term (GO6412) is represented by 26/67 proteins some of which, proteins involved in ribosomal machinery and an RNA binding protein (LinJ.32.0790), play role in post-transcriptional regulation of gene expression (∼18%, GO10608). Oxidation-reduction GO term is represented by cytochrome Cs (LinJ.07.0210, LinJ.16.1390), tryparedoxin peroxidase (LinJ.15.1100), tryparedoxin (LinJ.29.1250), and trypanothione reductase (LinJ.05.0350). Among proteins representing cellular macromolecular complex assembly GO term (GO34622) are ribosomal proteins and histone h3.

**Figure 7:**
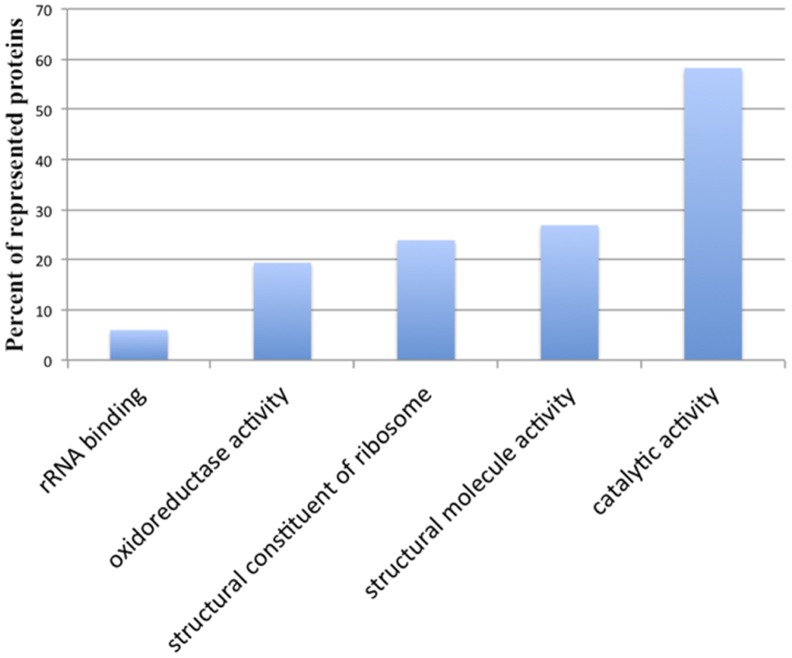
Molecular function GO term enrichment analysis. Significantly (*p* value < 0.05) over represented molecular functions are shown. P-values were determined by hypergeometric test after FDR correction. Percent of remodeled proteins represented in each GO term are shown in bars.

Five molecular function GO terms are significantly enriched in the modulated proteins (**Figure 7** **)**. The list of modulated proteins representing molecular function categories are given in **Table S5**. Catalytic activity (GO3824, 58%) is the most enriched followed by structural molecule activity (GO5198, 26.8%), structural constituent of ribosome activity (GO3735, 23.8%), oxido-reductase activity (GO16491, 19.4%) and rRNA binding activity (GO19843, 5.9%). Among 13 modulated proteins with oxido-reductase activity, all except for one (LinJ.34.0170, Malate dehydrogenase) are increased in the wild type parasites. All the proteins with ribosome structural constituent activity (GO3735) are increased in the wild type parasites. Only one of the modulated proteins with structural molecule activity (GO5198), alpha tubulin (TUB1, LinJ.13.1460), is increased in the null mutant parasites, and it is known to have structural role distinct from the 18 identified proteins with the same GO term. Among four proteins with rRNA binding activity (GO19843), only elongation factor 2 (LinJ.36.0210) is increased in the null mutant parasites and the remaining three are ribosomal proteins. The results indicate that significant number of proteins that are involved in oxido-reductase and structural constituents of ribosome activity are increased in cathepsin B wild type parasites.

**Figure 8:**
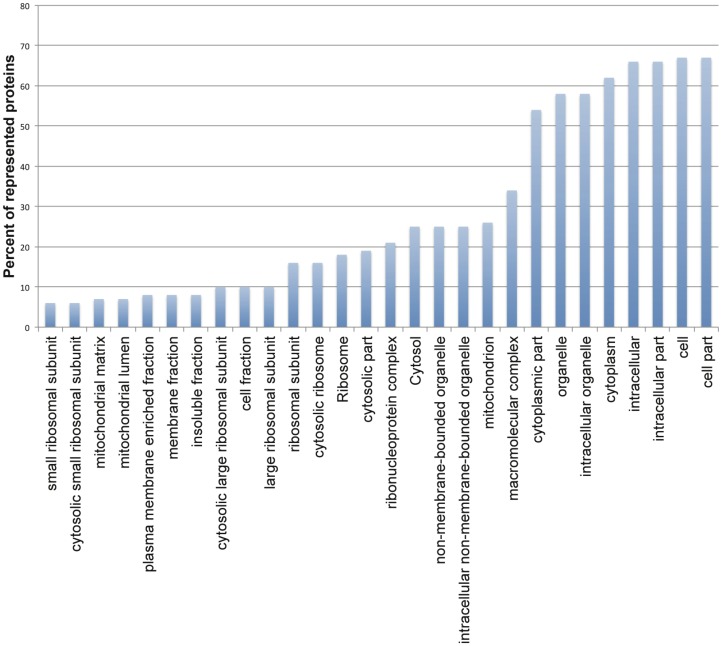
Cellular component GO term enrichment analysis. Significantly (*p* value < 0.05) over represented cellular components are shown. P-values were measured by hypergeometric test after FDR correction. Percent of remodeled proteins represented in each GO term are shown in bars. Non-redundant cellular components assigned to at least six (10%) remodeled proteins are displayed.

Enrichment analysis of the site of function of the modulated proteins indicates macromolecular complex (GO32991, 50.7%) and mitochondrion (GO-5739, 38.8%) among the most significantly enriched GO terms (**Figure 8**). The modulated proteins representing cellular component categories are given in **Table S6**. Among the enriched macromolecular complex structures are ribonucleoprotein complex (GO30529, 31.3%), respiratory chain complex III (GO45275, 3%) and proteasome core complex (GO9773, 3%). Proteins residing in the mitochondrion include those involved in oxido-reductase activity (GO16491) and cell death. Among proteins involved in cell death are cytochrome c1 (LinJ.07.0210, CyT1) and cytochrome c (LinJ.16.1390, CyT7). The cytochrome proteins are differentially modulated and unlike cytochrome c, cytochrome c1 is part of the respiratory chain complex III (GO45275).

### *L. donovani* proteins modulated by Cathepsin B gene disruption form molecular complex networks

**Figure 9:**
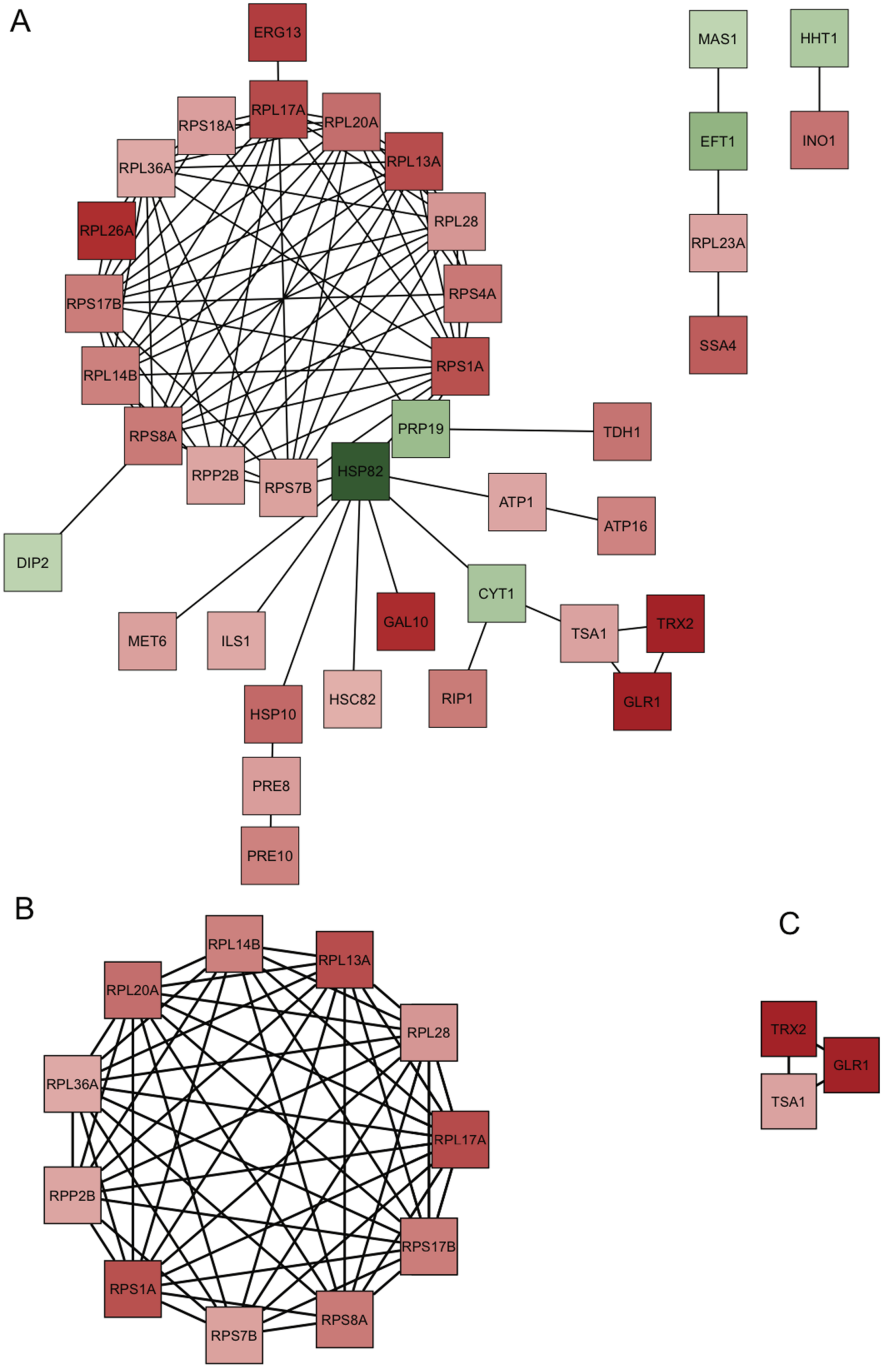
Network analysis of proteins modulated by cathepsin B gene disruption. Interaction network of 68 modulated proteins mapped to 67 yeast proteins (A). Highly connected network of ribosomal proteins (B). Highly connected network of oxidation-reduction proteins (C; TSA1, Peroxidoxin; GLR1, trypanothione reductase; TRX2, tryparedoxin). Proteins increased and decreased in the null mutant parasites are shown in green and red respectively. Node color intensity is proportional to the extent of modulation.

As shown above, cathepsin B gene disruption affects proteins with related function and those forming macromolecular complex structures. In order to gain more insight, we performed interaction network analysis on the modulated proteins **(** **Figure 9A** **).** Interaction analysis of the 67 proteins provided three different size networks made of 33, 4, and 2 nodes. More than 50% of the tested modulated proteins interact with one another and most of them belong to the same interaction network. The 33-node network consists of 28 nodes that were increased in the wild type parasites, whereas the 4-node and 2-node networks have an equal number of differentially modulated nodes. Ribosomal proteins **(** **Figure 9B** **)** and respiratory chain complex proteins **(** **Figure 9C** **)** form highly connected network of proteins increased in the wild type parasites.

## Discussion

We have generated and characterized *L. donovani* cathepsin B null mutant parasites by deleting part of cathepsin B coding region containing the active site cysteine residue and the occluding loop. Cathepsin B null mutants show significantly reduced activity against Z-Phe-Arg-AMC substrate indicating loss of cathepsin B activity. *L. donovani* cathepsin B null mutant parasites show ∼66% reduction in activity, whereas antisense cathepsin B inhibited *L. chagasi* had ∼35% reduction in activity [8]. We found no difference in the activity of *L. donovani* cathepsin B wild type and null mutant parasites against Z-Arg-Arg-AMC (data not shown). This is consistent with the predicted role of the S_2_ sub-site glycine residue in *L. donovani* and *L. mexicana* cathepsin B [12]–[14]. Therefore, like *T. brucei* cathepsin B, which happens to have a glycine residue at S_2_ sub-site, *L. major* and *L. donovani* cathepsin B may have indispensable role in survival and pathogenesis [11]. In agreement with previous study done by antisense inhibition of cathepsin B [8], *L. donovani* cathepsin B null mutants show significantly reduced virulence inside macrophages (**Figure 3**).

To gain insight on the mechanism of cathepsin B role in *Leishmania* survival and pathogenesis, the global effect of cathepsin B gene disruption on *L. donovani* proteome was studied by using iTRAQ based quantitative proteomics. We identified 463 proteins, of which 83 (18%) are modulated. We validated the proteome data by western blotting alpha tubulin and peroxidoxin-4 proteins. Most (66%) of the modulated proteins, including alpha tubulin and peroxidoxin, are *Leishmania* secreted proteins [32]. Some of these proteins, particularly those involved in protein turnover and cell death, are enriched in vesicle derived exoproteomes of *Leishmania* promastigotes [33]. *Leishmania* vesicle derived exosomes target virulence factors into host cell and modulate signalling pathways of macrophages to inhibit host defense system allowing the parasite to establish infection [34]–[36]. Hence, cathepsin B gene disruption induced modulation of secreted proteins and the significantly reduced virulence of the null mutants inside macrophage is consistent with the role of *Leishmania* secreted proteins. *Leishmania* secreted proteins potentiate anti-inflammatory immune response to suppress the host defense during infection [37]–[39]. Some of the modulated *Leishmania* secreted proteins, alpha-tubulin, ribosomal protein S4, and elongation factor 2, have been implicated in immune modulation [40]. We are currently investigating the effect of cathepsin B on the host immune system, and preliminary evidence suggests differential modulation of the host immune response by *L. donovani* cathepsin B wild type and null mutant parasites (unpublished data).

Significant number of proteins modulated by cathepsin B gene disruption play role in translation process (GO6412, 38.8%; **Table S4**). Most of these proteins are ribosomal proteins, which are increased in the wild type parasites and involved in post-transcription regulation of gene expression (GO10608; **Table S4).** *Leishmania* ribosomal proteins are stage-regulated with less protein level in amastigotes and believed to be responsible for decline of protein synthesis in the amastigotes [41]. They form highly connected interaction network (**Figure 9B** **)**, which are likely to be co-regulated. Recent findings in *Trypanosoma* suggest post-transcriptional regulon mediated co-regulation of functionally related proteins [42]–[45]. Post-transcriptional regulon mediated regulation of functionally related proteins and the effect of ribosomal proteins on protein synthesis remains to be determined in *Leishmania*. Post-transcriptional regulation of gene expression GO term is also represented by RNA binding protein, glycyl tRNA synthetase, and isoleucyl-tRNA synthetase (GO10608; **Table S4)**. Their role in post-transcriptional regulation of gene expression of *Leishmania* is unknown. Oxidation-reduction process (GO 55114, **Table S4**) is another significantly enriched GO term represented by proteins involved in cell death (cytochrome c and cytochrome c1) and in oxido-reductase activity (**Table 2**). Cytochrome c and c1 proteins are differentially modulated in cathepsin B wild type and null mutant parasites, and unlike cytochrome c, cytochrome c1 is part of the mitochondrial respiratory chain complex III (GO5750, **Table S4**). Cytochrome c1 cleavage by caspase 3 amplifies release of cytochrome c resulting in mammalian cell death [46]. Cathepsin B has been implicated in *Leishmania* cell death [47]. Since there are no caspases in *Leishmania*, the effect of cathepsin B on *Leishmania* cytochromes deserves an investigation. Most of the proteins with oxido-reductase activity are increased in cathepsin B wild type parasites (**Table 2**) and some are complex forming proteins (**Figure 9C**). Peroxidoxins or trypareodxin peroxidase, trypanothione reductase, and tryparedoxin are virulence factors of *Leishmania* known to have role in defense against oxygen and nitrogen free radicals [48]–[51]. Mitochondrial peroxidoxin (peroxidoxin 4) has been shown to prevent *Leishmania* cell death [52]. The decreased level of these proteins might explain the attenuated virulence of *L. donovani* cathepsin B null mutant parasites inside macrophages.

**Table 2:** Modulated proteins involved in oxido-reductase activity, translation, and proteolysis.

Id	Description	Log_2_(WT/KO)^a^	Δ^b^
**Proteins involved in oxido-reductase activity**			
LinJ.23.0050	Peroxidoxin	0.69	+
LinJ.05.0350	Trypanothione reductase	0.99	+
LinJ.25.1160	Aldehyde dehydrogenase, mitochondrial precursor	1.34	+
LinJ.15.1100	Tryparedoxin peroxidase	0.85	+
LinJ.15.1070	Glutamate dehydrogenase	1.25	+
LinJ.29.1250	Tryparedoxin	1.92	+
LinJ.34.0170	Mitochondrial malate dehydrogenase	–0.7	–
**Proteins involved in translation**			
LinJ.36.5870	isoleucyl-tRNA synthetase, putative	0.63	+
LinJ.35.1900	60S Ribosomal protein L36, putative	0.64	+
LinJ.35.3840	60S ribosomal protein L23, putative	0.67	+
LinJ.30.3790	60S acidic ribosomal protein P2, putative	0.68	+
LinJ.01.0440	ribosomal protein S7, putative	0.69	+
LinJ.30.3390	60S ribosomal protein L9, putative	0.7	+
LinJ.36.1000	40S ribosomal protein S18, putative	0.71	+
LinJ.36.4030	glycyl tRNA synthetase, putative	0.72	+
LinJ.35.3830	60S ribosomal protein L27A/L29, putative	0.78	+
LinJ.22.1410	40S ribosomal protein L14, putative	0.92	+
LinJ.28.2760	40S ribosomal protein S17, putative	0.96	+
LinJ.35.0600	60S ribosomal protein L18a, putative	1.06	+
LinJ.35.0410	40S ribosomal protein S3A, putative	1.3	+
LinJ.29.2580	60S ribosomal protein L13, putative	1.34	+
LinJ.32.0440	60S ribosomal protein L17, putative	1.35	+
LinJ.35.1670	60S ribosomal protein L26, putative	1.61	+
**Proteins involved in proteolysis**			
LinJ.05.0960	dipeptidyl-peptidase III	0.81	+
LinJ.27.0510	calpain-like cysteine peptidase	0.96	+
LinJ.29.0860	Cathepsin B	3.2	+
LinJ.35.1390	mitochondrial processing peptidase	–0.64	–
LinJ.21.2070	proteasome alpha 2 subunit	0.73	+
LinJ.27.0190	proteasome alpha 7 subunit	0.91	+

a Mean of log_2_ transformed wild type (WT) and knockout (KO) peptide ratios from two experiments. **^b^ Δ**, change in protein level; +, increased in WT; –, decreased in WT.

In summary, we have generated *L. donovani* cathepsin B null mutant parasites and revealed cathepsin B disruption induced modulation of *L. donovani* proteome. The results implicate cathepsin B in the regulation of *Leishmania* secreted proteins, particularly those involved in oxidation-reduction, cell death, and protein turnover. The generated null mutant parasites will be useful to elucidate the role of cathepsin B in *Leishmania* protein secretion and its effect on the host immune system *in vivo*.

## Supporting Information

Figure S1 **Cathepsin B amino acid sequence alignment.** LdCatB, LmajCatB, LmexCatB, HumanCatB, RatCatB and BovineCatB are *L. donovani*, *L. major*, *L. mexicana*, Human, Rat, and Bovine cathepsin B cysteine proteases respectively. The filled and unfilled arrows indicate pre and pro cleavage sites respectively. The Cysteine and Histidine active site residues are indicated with red arrows. Δ1, Δ2 and Δ3 represent deletion sites in *Leishmania* cathepsin B cysteine proteases. The red line underlines the occluding loop. The histidine residues of the occluding loop are shown with double green arrows. The conserved amino acid regions are colour shaded. The blue arrow indicates the S_2_ sub-site amino acids.
(TIFF)

Table S1 **Unique peptides used for the identification of** ***L. donovani*** **cathepsin B wild type and knock out proteome.** Among the 463 proteins identified, 459 proteins had at least two unique peptides that were detected in two independent experiments. The peptides corresponding to each protein are shown.
(XLS)

Table S2 **Relative abundance of** ***L. donovani*** **cathepsin B wild type and knock out proteins identified in two experiments.** *L. donovani* cathepsin B wild type (WT) and knock out (KO) protein ratios determined in two independent experiments (Exp. 1 and Exp. 2), mean of protein ratios of the two experiments, standard deviation of the means, and log_2_ transformed mean of the protein ratios are shown.
(XLS)

Table S3 ***L. donovani*** **proteins modulated by Cathepsin B gene disruption and their yeast top hits.** Accession number and gene description of the modulated proteins, their top yeast hits, and log2 transformed mean of their ratios in *L. donovani* wild type (WT) and knock out (KO) parasites are shown.
(XLS)

Table S4 **Enrichment analysis of biological process GO terms.** GO identification numbers (GO-IDs), terms associated with each GO-ID (Description), significance of each GO representations (*p-*Value), number of genes associated with each GO (Number of sequences), percentage of the genes associated (Percentage), and list of the genes (Genes) are shown.
(XLS)

Table S5 **Enrichment analysis of molecular function GO terms.** GO identification numbers (GO-IDs), terms associated with each GO-ID (Description), significance of each GO representations (*p-*Value), number of genes associated with each GO (Number of sequences), percentage of the genes associated (Percentage), and list of the genes (Genes) are shown.
(XLS)

Table S6 **Enrichment analysis of cellular component GO terms.** GO identification numbers (GO-IDs), terms associated with each GO-ID (Description), significance of each GO representations (*p-*Value), number of genes associated with each GO (Number of sequences), percentage of the genes associated (Percentage), and list of the genes (Genes) are shown.
(XLS)
