# Supplementary figures and images for: Cathepsin B Gene Disruption Induced Leishmania donovani Proteome Remodeling Implies Cathepsin B Role in Secretome Regulation
Source: PLoS One. 2013 Nov 14;8(11):e79951. doi: 10.1371/journal.pone.0079951 (PMC3828211; doi:10.1371/journal.pone.0079951)

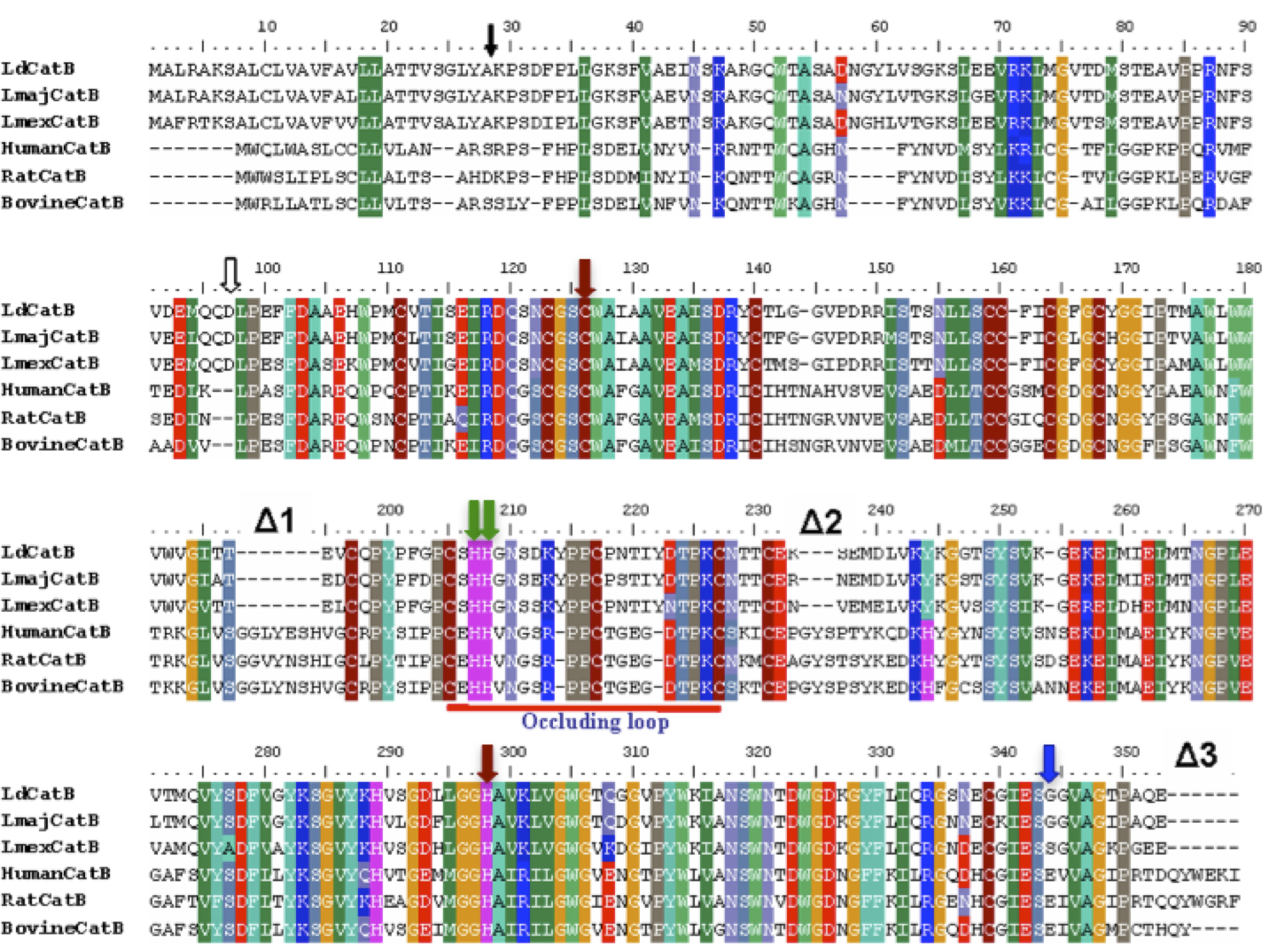

Supplement: Figure S1 — Cathepsin B amino acid sequence alignment. LdCatB, LmajCatB, LmexCatB, HumanCatB, RatCatB and BovineCatB are L. donovani, L. major, L. mexicana, Human, Rat, and Bovine cathepsin B cysteine proteases respectively. The filled and unfilled arrows indicate pre and pro cleavage sites respectively. The Cysteine and Histidine active site residues are indicated with red arrows. Δ1, Δ2 and Δ3 represent deletion sites in Leishmania cathepsin B cysteine proteases. The red line underlines the occluding loop. The histidine residues of the occluding loop are shown with double green arrows. The conserved amino acid regions are colour shaded. The blue arrow indicates the S2 sub-site amino acids. (TIFF) [file pone.0079951.s001.tiff]
